# Jasmonate Positively Regulates Cold Tolerance by Promoting ABA Biosynthesis in Tomato

**DOI:** 10.3390/plants12010060

**Published:** 2022-12-22

**Authors:** Fei Ding, Xizhi Wang, Ziye Li, Meiling Wang

**Affiliations:** School of Life Sciences, Liaocheng University, Liaocheng 252000, China

**Keywords:** *NCED*, *MYC2*, cold tolerance, tomato

## Abstract

As a cold-sensitive species, tomato is frequently challenged by cold stress during vegetative and reproductive growth. Understanding how tomato responds to cold stress is of critical importance for sustainable tomato production. In this work, we demonstrate that jasmonate (JA) plays a crucial role in tomato response to cold stress by promoting abscisic acid (ABA) biosynthesis. It was observed that both JA and ABA levels were substantially increased under cold conditions, whereas the suppression of JA biosynthesis abated ABA accumulation. The ABA biosynthesis gene *9-CIS-EPOXYCAROTENOID DIOXYGENASE2* (*NCED2*) was subsequently found to be associated with JA-mediated ABA biosynthesis in tomato plants in response to cold stress. *NCED2* was rapidly induced by exogenous MeJA and cold treatment. Silencing *NCED2* led to a decrease in ABA accumulation that was concurrent with increased cold sensitivity. Moreover, blocking ABA biosynthesis using a chemical inhibitor impaired JA-induced cold tolerance in tomato. Furthermore, *MYC2*, a core component of the JA signaling pathway, promoted the transcription of *NCED2*, ABA accumulation and cold tolerance in tomato. Collectively, our results support that JA signaling promotes ABA biosynthesis to confer cold tolerance in tomato.

## 1. Introduction

Tomato (*Solanum lycopersicum*) is well known as an economically important horticultural crop across the globe. Tomato fruits are well recognized as a source of multiple health-promoting nutrients, including lycopene and vitamins [1]. However, during tomato production, abiotic stresses, such as cold, heat, drought and salinity, often inhibit growth and development [2,3,4,5,6]. Cold is a major stress factor that poses great threat to growth, yields and quality in tomato, as this species originates from tropical or subtropical regions [7,8]. Thus, understanding how tomato responds to low-temperature conditions is a subject of substantial interest. A large number of studies have documented the deleterious effects of cold stress in plants. Reactive oxygen species (ROS) are notoriously accumulated under cold stress, eventually leading to oxidative damage to a plethora of macromolecules, such as proteins, nucleic acids and sugars [9,10,11,12]. Cold stress also impairs photosynthesis by inactivating major enzymes that participate in the process of carbon fixation [13]. For example, sedoheptulose-1,7-bisphosphatase, which is a crucial enzyme for the production of ribulose-1,5-bisphosphate in carbon fixation, undergoes a rapid loss of activity under cold conditions [14].

As sessile organisms, plants have evolved sophisticated mechanisms at the physiological, cellular, and molecular levels to cope with low-temperature conditions. At the physiological level, for instance, upon cold stress, plants synthesize and accumulate numerous protective substances and proteins, such as soluble sugars, proline, and LEA proteins [15,16,17]. At the molecular level, several critical genes play a role in the cold response, among which *CBF* genes are best characterized and play central roles in the transcriptional regulatory network under low-temperature conditions [18,19,20]. CBF transcription factors enhance cold tolerance by targeting and promoting the expression of numerous cold-regulated genes, such as *COR*, *KIN* and *LTI* [20,21,22]. Phytohormones also act as central players during the adaptation to cold conditions by coordinating numerous biological processes. Jasmonates (JAs), abscisic acid (ABA), ethylene (ET), salicylic acid (SA) and brassinosteroid (BR) positively regulate cold tolerance [23,24,25,26,27,28], whereas cytokinin (CK) plays a negative role in cold tolerance [29].

JAs are composed of jasmonic acid (JA), jasmonyl isoleucine (JA-Ile) and methyl jasmonate (MeJA), among others. JAs have been well recognized as defense hormones that protect plants against pathogen attack and insect herbivory [30,31]. Recently, JAs have emerged as crucial players in the cold response in plants. The rising levels of JAs upon cold stress have been observed in different species, including tomato, Arabidopsis, rice and *Artemisia annua* [32,33,34,35], indicating that JAs are involved in the tolerance to cold stress. The role of JAs in the regulation of the cold response is further supported by experimental evidence that exogenous JAs increase the cold tolerance of orange, banana and tomato [36,37,38]. Furthermore, molecular studies have shown that JAs increase cold tolerance by activating the ICE-CBF module. JAZs, negative regulators of JA signaling, directly interact with ICEs and suppress their transcriptional activity on CBFs. Cold-induced accumulation of JAs initiates JAZ degradation, thus releasing ICEs and activating CBFs [33]. Additionally, JAs have been demonstrated to improve cold resistance by activating the biosynthesis of betaine and putrescine in a *MYC2*-dependent manner in plants [35,39]. Interestingly, JA signaling targets a glutathione *S*-transferase to mitigate oxidative stress triggered by cold stress in tomato [38]. More recently, two studies have demonstrated that JAs mediate the cold response in tomato by crosstalk with melatonin and ethylene [28,40].

ABA is another phytohormone that is important for the stress response in plants. It is also a central player in the trade-off between growth and stress response [41,42]. The pathways for ABA biosynthesis and signaling have now been well established. In ABA biosynthesis, several major enzymes are involved, including zeaxanthin epoxidase, alcohol dehydrogenase, abscisic aldehyde oxidase and 9-*cis*-epoxycarotenoid dioxygenase (NCED) [43,44,45]. The production of the ABA precursor xanthoxin relies on the catalytic activity of NCED, which determines the overall rate of ABA biosynthesis [46,47]. Upon perception of ABA, ABA receptors PYRABACTIN RESISTANCE1 (PYR1)/PYR1-like (PYL) release SnRK2, which is suppressed by PP2Cs. SnRK2s then activate downstream ABA transcription factors via phosphorylation. ABA has been reported to be crucial in plant responses to cold stress in a growing number of studies. Recently, ABA has been found to interact with other hormones, such as strigolactones and brassinosteroids, to positively regulate cold tolerance in tomato [48,49]; however, it remains largely unknown whether ABA and JA interact in tomato when exposed to cold stress.

As both JA and ABA have been implicated in the cold response in plants, we hypothesized that their crosstalk is critical for cold tolerance in tomato. Thus, the goal of this study was to clarify the relationship between JA and ABA during tomato responses to cold stress and identify key genes that are involved in the interaction of JA and ABA. The results of this work will advance our understanding of the crosstalk between JA and ABA in tomato when exposed to cold stress.

## 2. Results

### 2.1. JA and ABA Accumulate in Tomato Leaves in Response to Cold Stress

**Figure 1:**
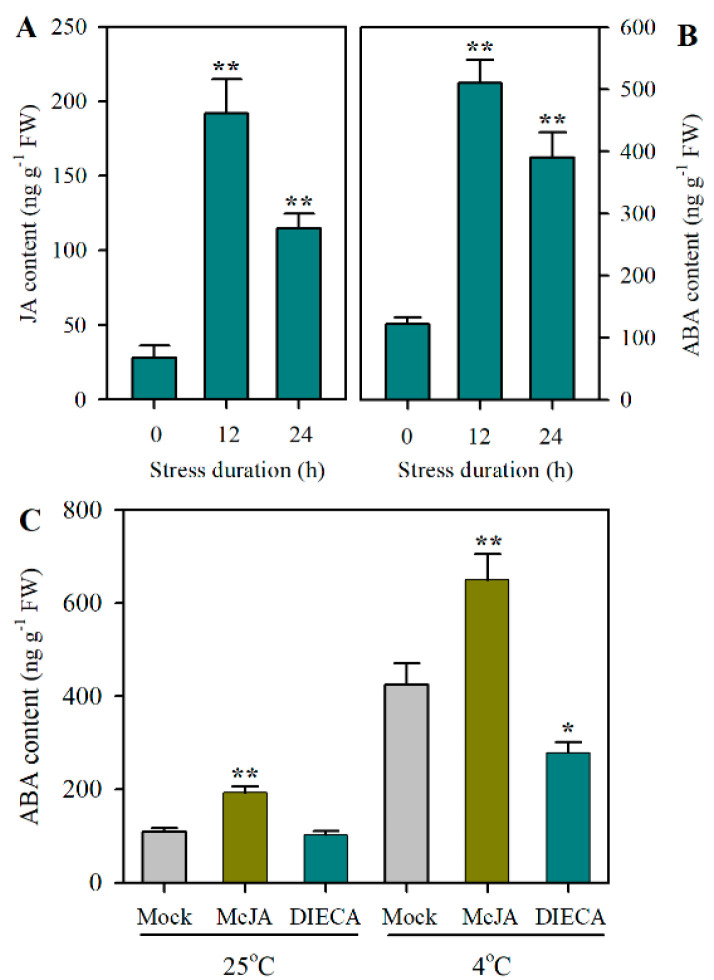
Accumulations of JA and ABA in tomato leaves under cold stress. (**A**) JA levels; (**B**) ABA levels; (**C**) changes in ABA levels as affected by exogenous MeJA and DIECA. Tomato plants at the 4-leaf stage were subjected to cold stress at 4 °C for 24 h. Leaf samples collected at 12 h and 24 h following cold stress were used for determination of accumulations of endogenous JA and ABA. Exogenous MeJA and DIECA were applied 12 h prior to cold treatment and ABA levels were determined following 24 h of cold stress. Data presented are means of three biological replicates (±SD). Asterisks indicate significant differences (** *p* < 0.01, * *p* < 0.05) according to Student’s *t*-test. FW, fresh weight; MeJA, methyl jasmonate; DIECA, sodium diethyldithiocarbamate.

To understand the potential crosstalk between JA and ABA in tomato plants exposed to cold stress, we first investigated the levels of JA and ABA. It was observed that at 12 h and 24 h following cold treatment, the accumulation of JA was increased by 580% and 310%, respectively (Figure 1A). Similarly, ABA production was substantially increased by cold stress, with the ABA level being enhanced by 318.0% at 12 h and 220% at 24 h after cold stress was imposed (Figure 1B). Intriguingly, under cold stress, MeJA substantially elevated the accumulation of ABA in comparison with the mock treatment, whereas sodium diethyldithiocarbamate (DIECA), a JA biosynthesis inhibitor, attenuated cold-triggered accumulation of ABA (Figure 1C). These observations imply that both JA and ABA may be involved in the tomato cold response and JA may interact with ABA by modulating its accumulation under cold stress.

### 2.2. Identification of JA-Responsive ABA Biosynthesis Gene NCED2 under Cold Stress

After observing that JA was involved in ABA accumulation under cold stress, we next attempted to identify JA-targeted ABA biosynthesis genes in tomato. By analyzing previously published transcriptome data, we identified a JA-responsive gene *NCED2*, which is known to play a key role in tomato ABA biosynthesis. Sequence analysis displayed that the transcript length of *NCED2* was 1746 bp, which encoded a predicted protein of 581 amino acids. Gene structural analysis revealed that *NCED2* contained only one exon. NCED2 has an estimated molecular weight of 64.83 kDa and a predicted isoelectric point of 6.51.

**Figure 2:**
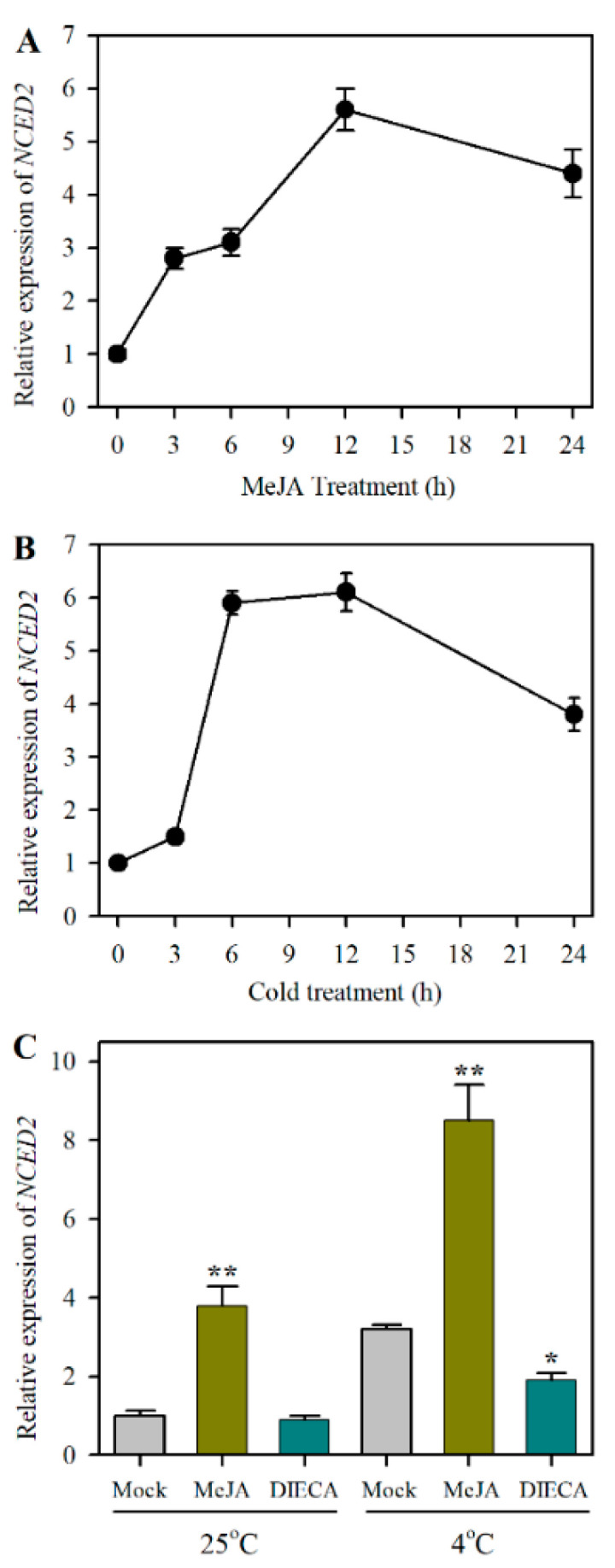
Expression of *NCED2* in response to JA and cold stress. (**A**) Time course of *NCED2* expression in response to MeJA treatment. (**B**) Time course of *NCED2* expression during a 24 h cold treatment. (**C**) Changes in *NCED2* expression as affected by exogenous MeJA and DIECA under cold stress. Tomato leaves were detached and floated on 50 µM MeJA for 24 h. Leaf samples were collected at indicated time points and were used for the expression analysis of *NCED2* by qRT-PCR. Tomato plants at the 4-leaf stage were subjected to cold stress at 4 °C for 24 h and leaf samples were collected at indicated time points for the expression analysis of *NCED2* by qRT-PCR. Exogenous MeJA and DIECA were applied to tomato leaves 12 h prior to the cold treatment and the expression of *NCED2* was determined by qRT-PCR following 24 h of cold stress. Data presented are means of three biological replicates (±SD). Asterisks indicate significant differences (** *p* < 0.01, * *p* < 0.05) according to Student’s *t*-test.

We then examined the time course of *NCED2* expression in response to MeJA to verify the association of JA with *NCED2*. Tomato leaves were detached and incubated in MeJA solution for 24 h and the relative expression of *NCED2* was assessed by qRT-PCR. During MeJA treatment, *NCED2* expression was rapidly induced at 3 h and peaked at 12 h (Figure 2A). After establishing that JA induced the expression of *NCED2*, we next asked whether cold stress also triggered *NCED2* expression. To seek an answer, we subjected tomato plants (four-leaf stage) to cold stress (4 °C) for 24 h. It was shown that cold stress led to a remarkable increase in the expression of *NCED2*, with the transcript level being increased by 5.9-fold at 5 h following cold treatment (Figure 2B). These results suggest that *NCED2* is responsive to both JA and cold stress.

To further verify the relationship between JA and ABA biosynthesis genes in response to cold stress, we performed pharmacological studies using exogenous MeJA and DIECA, which is a commonly used inhibitor of JA biosynthesis. qRT-PCR analysis revealed that under cold stress, the application of MeJA markedly promoted the expression of *NCED2*, while DIECA suppressed the expression of *NCED2*, compared with the mock treatment (Figure 2C). Under optimal growth conditions, MeJA enhanced the transcripts of *NCED2*, whereas DIECA did not significantly alter the transcripts of *NCED2* in comparison with the mock treatment. These results were consistent with the ABA quantification following treatments of MeJA and DIECA under cold stress (Figure 1C), suggesting that JA promotes ABA accumulation likely by inducing the expression of *NCED2* in tomato challenged by cold stress.

### 2.3. Suppression of NCED2 Attenuates Cold Tolerance in Tomato

**Figure 3:**
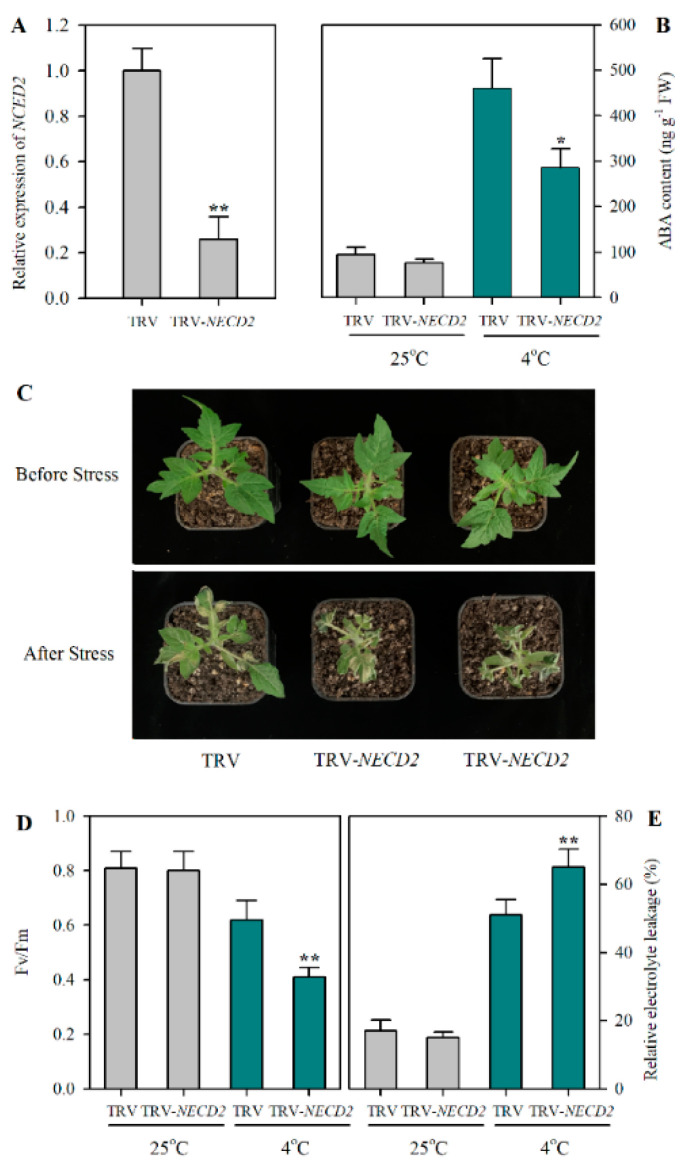
Effects of *NCED2* suppression on cold tolerance in tomato. (**A**) Relative expression of *NCED2* in TRV and TRV-*NCED2* plants under cold conditions. (**B**) ABA contents in TRV and TRV-*NCED2* plants under cold conditions. (**C**) Phenotypes of TRV and TRV-*NCED2* tomato plants subjected to cold stress. (**D**) Fv/Fm in TRV and TRV-*NCED2* plants under cold conditions. (**E**) Relative electrolyte leakage in TRV and TRV-*NCED2* plants under cold conditions. TRV and TRV-*NCED2* tomato plants were exposed to cold stress and leaves were collected for the expression analysis of *NCED2* and cold tolerance assays. Data presented are means of three biological replicates (±SD). Asterisks indicate significant differences (** *p* < 0.01, * *p* < 0.05) according to Student’s *t*-test.

As *NCED2* was associated with the tomato cold response, we continued to explore how it might affect tomato cold tolerance. We suppressed the expression of *NCED2* via virus-induced gene silencing (VIGS) and plants with reductions in *NCED2* transcript abundance by ~80% were selected for analyses of ABA levels and cold tolerance (Figure 3A). Under cold stress, *NCED2*-silenced plants accumulated remarkably less ABA than the control plants, while under optimal growth conditions, *NCED2*-silenced plants did not show any significant differences compared to the control plants in the accumulation of ABA (Figure 3B). Phenotype analysis showed that VIGS plants displayed more cold damage than the control plants (Figure 3C). Moreover, silencing *NCED2* led to a decrease in Fv/Fm (Figure 3D), which is a commonly used indicator of cold tolerance. Additionally, *NCED2*-silenced plants demonstrated higher relative electrolyte leakage (REL) than the control plants (Figure 3D). These observations imply that *NCED2* positively regulates cold tolerance by increasing ABA accumulation in tomato plants under cold stress.

### 2.4. ABA Mediates JA-Induced Cold Tolerance

**Figure 4:**
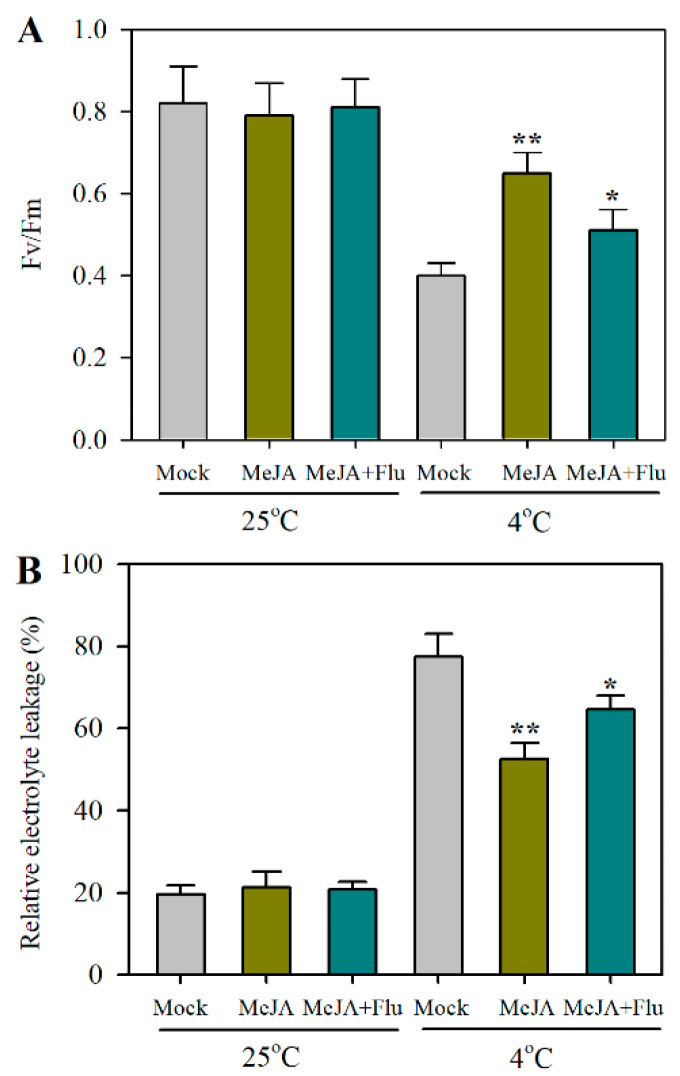
ABA contributes to JA-induced cold tolerance in tomato. (**A**) Changes in Fv/Fm as affected by MeJA and the ABA biosynthesis inhibitor fluridone (Flu) under cold conditions. (**B**) Changes in relative electrolyte leakage as affected by MeJA and the ABA biosynthesis inhibitor fluridone (Flu) under cold conditions. Tomato plants at the 4-leaf stage were treated by MeJA and Flu 12 h prior to cold stress at 4 °C for 24 h. Cold tolerance was assessed by determining Fv/Fm and relative electrolyte leakage. Data presented are means of three biological replicates (±SD). Asterisks indicate significant differences (** *p* < 0.01, * *p* < 0.05) according to Student’s *t*-test.

We found that JA induced the expression of *NCED2* and *NCED2* positively regulated cold tolerance by increasing ABA accumulation, which prompted us to hypothesize that ABA mediates JA-induced cold tolerance in tomato. To test this hypothesis, we took advantage of the ABA biosynthesis inhibitor fluridone (Flu) to partially block ABA accumulation in tomato leaves. It was shown that cold stress adversely affected the maximal photochemical efficiency of PSII, as reflected by the substantial decrease in Fv/Fm, while exogenously applied MeJA considerably alleviated cold-induced suppression of Fv/Fm. MeJA-treated plants displayed a higher maximal photochemical efficiency of PSII than the mock plants under cold conditions, with Fv/Fm being increased by 58.8%. However, the application of Flu attenuated the effects of MeJA on Fv/Fm. Compared with the MeJA treatment, the Flu treatment decreased Fv/Fm by 21.6% (Figure 4A). The assessment of relative electrolyte leakage revealed that under cold stress, MeJA significantly reduced the relative electrolyte leakage, while the addition of Flu weakened the effects of MeJA, leading to increased electrolyte leakage (Figure 4B). The results demonstrate that JA promotes cold tolerance, depending partially on ABA.

### 2.5. JA Promotes ABA Biosynthesis via MYC2-Regulated Transcription of NCED2 under Cold Stress

**Figure 5:**
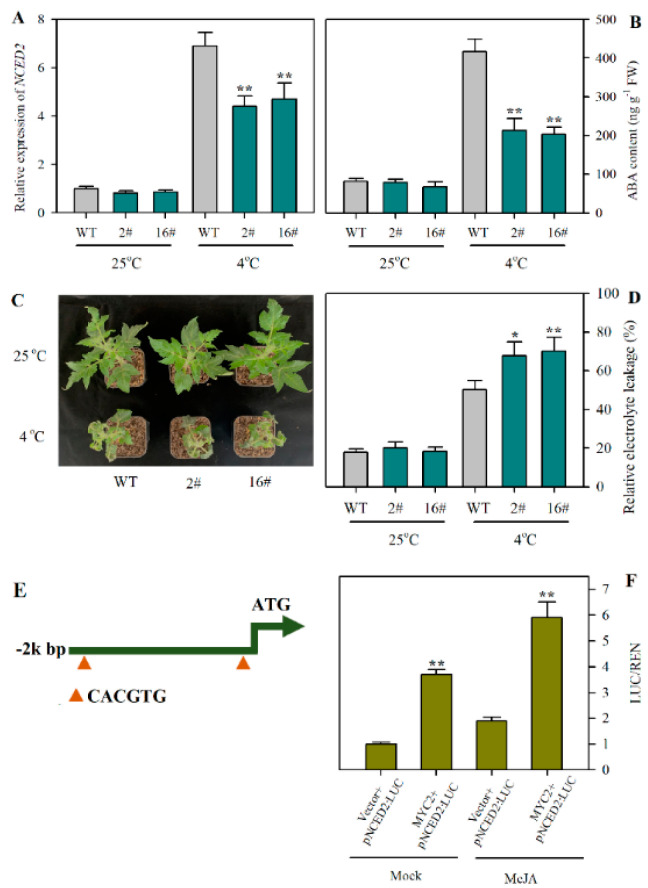
*MYC2* regulates the transcription of *NCED2* and cold tolerance in tomato. (**A**) Alterations in the relative expression of *NCED2* as affected by the suppression of *MYC2* in tomato under cold stress. (**B**) Alterations in ABA content as affected by the suppression of *MYC2* in tomato under cold stress. (**C**) Phenotypes of *MYC2*-RNAi plants under cold stress. (**D**) Relative electrolyte leakage in *MYC2*-RNAi plants under cold stress. (**E**) Predicted binding sites of *MYC2* in the promoter region of *NCED2*. (**F**) Dual-luciferase analysis of *MYC2* binding to *NCED2* promoter. Relative activity is shown by LUC/REN. Data presented are means of three biological replicates (±SD). Asterisks indicate significant differences (** *p* < 0.01, * *p* < 0.05) according to Student’s *t*-test.

As *MYC2* acts as a crucial regulator of the JA signaling pathway, we next examined if JA-induced *NCED2* expression and ABA accumulation were associated with *MYC2*. We utilized previously generated *MYC2*-RNAi plants to investigate the function of *MYC2* in the transcription of *NCED2* under cold stress. It was observed that under cold conditions, the suppression of *MYC2* led to a decrease in the relative expression of *NCED2* by 36.2% and 31.9% in transgenic lines 2# and 16#, respectively (Figure 5A). Concomitantly, the accumulations of ABA in *MYC2*-RNAi lines were significantly reduced following cold stress (Figure 5B). The cold tolerance assay showed that suppression of *MYC2* resulted in decreased cold tolerance, as evidenced by the cold-sensitive phenotype and enhanced relative electrolyte leakage (Figure 5C,D).

In an attempt to further unravel the regulatory role of *MYC2* in the transcription of *NCED2*, we took a closer look at the *NCED2* promoter. We analyzed the 2k promoter region using PlantCare [50] and identified two G-box elements that have been reported as *MYC2* binding sites (Figure 5E), which are suggestive of the possible association of *MYC2* with the *NCED2* promoter. Furthermore, we performed dual-luciferase assays to verify the transactivation activity of *MYC2* on the *NCED2* promoter. It was shown that *MYC2* activated the expression of *NCED2*, which was inferred from the increased promoter activity, as indicated by the enhanced LUC/REN (Figure 5F). These results clearly demonstrate that *MYC2* positively regulates the transcription of *NCED2* and ABA accumulation in tomato cold stress.

## 3. Discussion

As a major abiotic stress, low temperature inhibits plant growth and causes considerable losses of crop yields. Tomato is a warm-climate species and cold stress adversely affects its growth and development at nearly all stages, including vegetative, reproductive, and fruiting. It is, thus, critical to understand how tomato responds to cold stress at the physiological and molecular levels. Initially identified as a defense hormone against pathogen and insect attack, JAs have recently emerged as crucial players in adaptation to cold stress in plants. The positive role of JAs in cold tolerance has been observed in a variety of species, such as Arabidopsis [33], banana [36], rice [51], trifoliate orange [39] and tomato [35]. Several mechanisms for JA-mediated cold tolerance have been proposed in these studies; however, our knowledge on the mechanisms by which JA regulates cold response is still rudimentary, in particular in tomato. ABA is a well-known phytohormone that is vital for a plethora of abiotic stress responses, including cold response [52,53]. Previous studies have established the crosstalk between JA and ABA during stress responses [42,54]; however, whether and how these two hormones interact to regulate tomato cold response are still unclear. In this work, we determined that JA promotes tomato cold tolerance by upregulating the ABA biosynthesis gene *NCED2*. We showed that *NCED2* was responsive to both cold stress and JA and silencing *NCED2* resulted in decreased ABA accumulation and increased cold sensitivity in tomato. Furthermore, blocking ABA biosynthesis attenuated JA-induced cold tolerance. We also revealed that the JA-triggered expression of *NCED2* partially relied on *MYC2*, which is a core regulator of the JA signaling pathway.

Previous reports have pointed to the importance of JA in the tomato cold response. In this study, we observed that JA accumulation was enhanced under cold stress, substantiating the involvement of JA in the tomato cold response. Intriguingly, JA appears to be related with ABA in response to cold conditions in tomato, as the accumulation of JA was concurrent with that of ABA, with both peaking at 12 h following cold treatment. The association of JA with ABA in the tomato cold response was further supported by the applications of exogenous MeJA and DIECA, a biosynthesis inhibitor of endogenous JA, under cold conditions. Our data herein showed that whereas MeJA promoted cold-triggered ABA accumulation, DIECA attenuated cold-induced ABA accumulation in tomato, implying the crosstalk between JA and ABA. Similar relations between JA and ABA were also observed in drought-stressed Arabidopsis plants [55]. However, contrary to what we observed in this study, a previous work reported that a decreased level of JA did not exert a pronounced effect on the accumulation of ABA in tomato under drought conditions [56]. Together, these results suggest the complexity of the crosstalk between JA and ABA, which may be determined by the type of stresses and species.

Our observation that JA promoted ABA accumulation raises an interesting question of how JA acts in the biosynthesis of ABA under cold stress. It has been established that NCED functions as a rate-limiting enzyme during ABA biosynthesis [43,57]. The genes that encode NCED usually form a multigene family. In tomato, three NCED genes, including *NCED1*, *NCED2*, and *NCED6*, have been identified to play major roles in ABA biosynthesis [58,59]. We, thus, hypothesized that *NCED* might be targeted by JA. To validate our hypothesis, we performed transcriptome analysis in an attempt to identify JA-targeted *NCED* genes. *NCED2* was identified to be upregulated by JA. The regulation of *NCED2* transcription by JA was further corroborated by our time-course analysis of *NCED2* expression upon MeJA treatment, in which the transcript abundance of *NCED2* was remarkably elevated. It is worth nothing that a previous study demonstrated that JA deficiency led to increased *NCED1* transcript accumulation in tomato in response to water stress [56]. Interestingly, another report shows that JA induced the expression of *NCED3* to regulate stomatal closure [60]. Thus, our results and those of previous studies highlight that JA is crucial for the regulation of ABA biosynthesis genes in response to environmental stress.

*NCED* genes have been previously shown to be responsive to salinity, drought and cold. After establishing that JA positively regulates the expression of *NCED2*, we next sought to understand the role of JA-*NCED2* in tomato under cold conditions. Our time-course examination of *NCED2* expression in tomato leaves under cold stress revealed that *NCED2* was responsive to cold stress and was rapidly induced, implying that *NCED2* may be involved in the tomato cold response. Here, we found that the silencing of *NCED2* via VIGS led to enhanced cold sensitivity in tomato, as evidenced by the decreased Fv/Fm and increased REL levels. The altered cold sensitivity may be associated with the change in the level of endogenous ABA, as ABA accumulation was markedly reduced in *NCED2* VIGS plants compared with that in control plants under cold stress. Furthermore, exogenous MeJA significantly promoted cold tolerance, while exogenous fluridone (Flu), an ABA biosynthesis inhibitor, abated the effects of MeJA on cold tolerance.

As a core component of the JA signaling pathway, *MYC2* has been verified to play essential roles in multiple developmental processes and stress responses. Whether *MYC2* is involved in JA-induced *NCED2* expression and ABA accumulation under cold stress remains unanswered. In the current work, multiple lines of evidence support the role of *MYC2* in regulating *NCED2* expression, ABA accumulation, and cold tolerance in tomato. First, the suppressed expression of *MYC2* in *MYC2*-RNAi plants led a reduction in *NCED2* in tomato under cold stress. Second, two potential *MYC2* binding sites were identified in the 2 kb promoter region of *NCED2*. Third, dual-luciferase assays demonstrated that *MYC2* activated the transcription of *NCED2*. Lastly, in accordance with decreased *NCED2* expression, ABA accumulation was largely reduced in *MYC2*-RNAi tomato plants under cold stress. Consistently, *MYC2*-RNAi tomato plants also demonstrated decreased cold tolerance, as indicated by the increased REL in tomato leaves following cold treatment. Hence, *MYC2* acts as a crucial regulator in JA-triggered ABA accumulation and cold resistance in tomato.

## 4. Materials and Methods

### 4.1. Plant Materials and Growth Conditions

Three genotypes of tomato (*Solanum lycopersicum* ‘MicroTom’), including wild-type tomato, transgenic tomato (*MYC2*-RNAi) and VIGS tomato (TRV-*NCED2*), were used as plant materials in this work. Tomato seedlings were grown in substrate that contained peat and vermiculite (2/1, *v*/*v*) in a growth chamber, with the following growth conditions: 14 h light/8 h dark cycles with the light density of 200 µmol m^−2^ s^−1^, 26 °C/21 °C (light/dark), and ∼70% relative humidity.

### 4.2. Cold Treatments

For the cold treatments, tomato plants were exposed to 4 °C at the 4-leaf stage. To determine the JA and ABA accumulations, cold stress was imposed on the tomato plants for 24 h. To determine the relative expression of *NCED2* in response to MeJA, young leaves were detached and subjected to MeJA treatment by floating on 50 µM of MeJA solution in petri dishes for 24 h. To determine the relative expression of *NCED2* in response to cold stress, tomato seedlings were subjected to cold stress for 24 h and leaf samples were collected at 0, 3, 6, 12 and 24 h following cold treatment. To understand the importance of JA in *NCED2* expression and ABA accumulation under cold stress, we first sprayed tomato plants at the 4-leaf stage with mock solution, MeJA (100 µM), or DIECA (2 mM), and 12 h later, these plants were subjected to cold stress. To understand how *MYC2* might regulate the expression of *NCED2*, *MYC2*-RNAi tomato plants at the 4-leaf stage were grown under cold conditions for 24 h.

### 4.3. Assessment of Cold Tolerance

Two cold-sensitive parameters, Fv/Fm and REL (relative electrolyte leakage), were measured to determine the cold tolerance of tomatoes. Fv/Fm measurements were performed following the procedures reported previously [8]. After cold treatment, to obtain the minimal fluorescence (Fo), we placed tomato plants in the dark for 30 min and following dark adaptation, Fo was measured with a portable chlorophyll fluorometer. After completing measurements of Fo, we applied a saturating pulse to the leaves to obtain the maximal fluorescence (Fm).

REL was measured as previously reported [38]. In brief, after cold treatment, tomato leaves were detached and incubated in deionized water to obtain the initial conductivity (C1). Leave materials were subsequently boiled to obtain the maximum conductivity (C2). REL was expressed as C1/C2 × 100%.

### 4.4. Expression Analysis of NCED2

qRT-PCR was performed to quantify the relative transcript abundance of *NCED2*. Briefly, we first isolated the total RNA using an RNAprep Pure Plant Kit (TIANGEN, Beijing, China). Then, we synthesized cDNA using the total RNA as a template. PCR reactions were carried out with a commercial kit (Premix Ex Taq, TaKaRa Bio, Dalian, China). *ACTIN2* in tomato was used as an internal control. All measurements were performed with two technical and three biological replicates. The primers for relative gene expression analysis of *NCED2* were GAGGACGATGGATATATTCTTGCA (forward) and AGTCCTTTGAGCTAATGAATGTCC (reverse). The primers of *ACTIN2* used for qRT-PCR analysis were TCCCAGCAGCATGAAGATTAAG (forward) and CCTGTGGACAATGGATGGAC (reverse).

### 4.5. Quantification of JA and ABA

Accumulations of JA and ABA in tomato leaves after cold treatments were measured according to a previous study [28]. To extract JA and ABA, we first ground the leaves to a fine powder with liquid N_2_, which was then homogenized in ethyl acetate. Ethyl acetate was spiked with D6-ABA and D5-JA, which acted as internal standards. The homogenate was subsequently centrifuged at 18,000× *g* for 10 min at 4 °C. Then, the supernatant was collected and evaporated to dryness under nitrogen gas. We resuspended the pellets in methanol (70%, *v*/*v*) and centrifuged them at 18,000× *g* for 2 min. The supernatants were collected and subjected to HPLC–mass spectrometry analysis. Endogenous JA and ABA levels were expressed as ng/g fresh leaves.

### 4.6. Virus-Induced Gene Silencing (VIGS) of NCED2

To knock down the expression of *NCED2*, we employed virus-induced gene silencing using tobacco rattle virus (TRV) vectors, TRV1 and TRV2, according to a previous study [12]. A fragment (300 bp) of *NCED2* was selected using the SGN VIGS Tool. The fragment was then amplified and ligated into the TRV2 vector, and an empty TRV2 vector was used as a control. We then introduced all the constructs into *Agrobacterium tumefaciens* GV3101. Cultures of *Agrobacterium tumefaciens* with TRV1, TRV2-*NCED2*, or TRV2 were mixed and the mixture was used to inoculate fully expanded cotyledons. The infected seedlings were maintained in a growth chamber for four weeks, and then leaves were collected for qRT-PCR analysis of *NCED2* expression. Plants with ~80% reductions in *NCED2* expression were selected for further experiments.

### 4.7. Dual-Luciferase Assays

We performed dual-luciferase assays using tobacco (*Nicotiana benthamiana*) leaves, as described in a previous study [40]. Two vectors, including pGreen II 0029 62-SK (SK) and pGreen II 0800-LUC (LUC), were used for the dual-luciferase assays. We first amplified the full-length coding fragment of *MYC2* and ligated it to the pGreen II 0029 62-SK (SK) vector to produce the effector construct. Then, we cloned the promoter fragment (2 kb) of *NCED2* and ligated it into the pGreen II 0800-LUC (LUC) vector to generate the reporter construct. Both constructs were then introduced into the *Agrobacterium tumefaciens* strain GV3101. We then mixed cultures of *Agrobacterium tumefaciens* that harbored the effector construct or the reporter construct and used the mixture to inoculate tobacco leaves. A Dual-Luciferase Assay Kit (Promega, Madison, WI, USA) was used to measure the LUC and REN activities three days after inoculation, and the ratio of LUC/REN was calculated.

## 5. Conclusions

**Figure 6:**
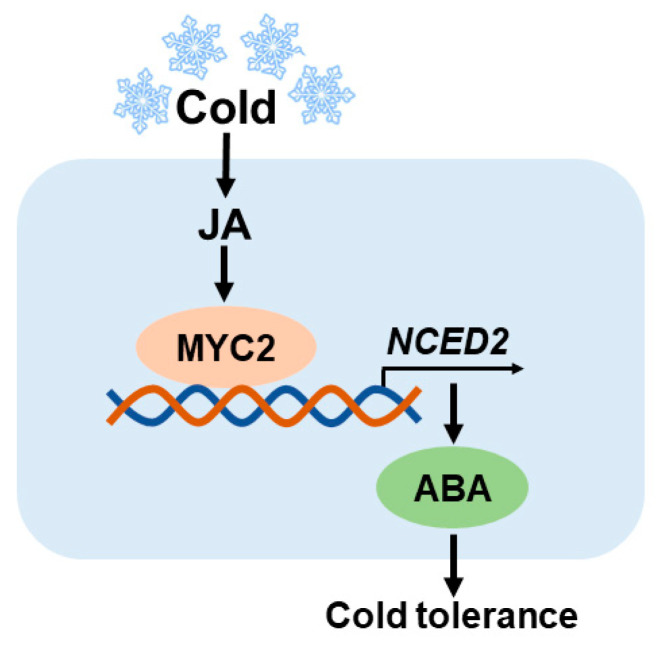
Simplified model that depicts how jasmonate regulates ABA biosynthesis in response to cold stress. Upon cold stress, JA accumulation is increased in tomato, initiating JA signaling and activating *MYC2*. *MYC2* acts on the promoter of ABA biosynthesis gene *NCED2* and promotes its transcription, leading to increased accumulation of ABA. Thus, JA-mediated cold tolerance is partially dependent on JA-induced ABA biosynthesis in tomato.

In summary, we demonstrate the crosstalk between JA and ABA at the biosynthetic level in tomato leaves under cold stress. JA triggers the expression of the ABA biosynthesis gene *NCED2* through *MYC2*, eventually promoting ABA accumulation and cold resistance in tomato. Finally, we propose a working model of JA-mediated cold tolerance via ABA biosynthesis (Figure 6). This work expands our understanding of JA-mediated cold tolerance and the crosstalk between JA and ABA in plants under stress conditions.
